# A Novel Predictive Marker for In-Hospital Mortality in Acute Cerebral Infarction: Low-Density Lipoprotein Cholesterol to Lymphocyte Ratio

**DOI:** 10.7759/cureus.9986

**Published:** 2020-08-24

**Authors:** Muzaffer Güneş, Hüseyin Büyükgöl

**Affiliations:** 1 Neurology, Aksaray University Training and Research Hospital, Aksaray, TUR; 2 Neurology, KTO Karatay University, Konya, TUR

**Keywords:** acute cerebral infarction, low density lipoprotein-cholesterol, lymphocyte, stroke, high density lipoprotein-cholesterol, red blood cell distribution width

## Abstract

Objectives

Neutrophil to eosinophil ratio, neutrophil to lymphocyte ratio, C-reactive protein to albumin ratio, and red blood cell distribution width (RDW) have been studied in patients with acute cerebral infarction (ACI). However, the low-density lipoprotein cholesterol (LDL-C) to lymphocyte ratio has never been studied. Hence, our objective was to study the LDL-C to lymphocyte ratio with regard to in-hospital mortality rates of patients with ACI.

Materials and methods

We retrospectively examined our patients diagnosed with ACI between January 2015 and December 2018. The patients' clinical data and imaging and laboratory results during the acute period were retrieved from our database and saved for statistical analysis. The receiver operating characteristic (ROC) curve analysis was performed to evaluate the predictive value of the variables and to calculate the cut-off values.

Results

A total of 172 patients with ACI, including 43 patients who died and 129 patients who survived in the hospital, were included in the study. The median age of the patients who died was significantly higher than that of those who survived (p: <0.001). Median triglyceride level, LDL-C to lymphocyte ratio, and RDW-SD values were significantly higher in patients who died (p = 0.017, p: <0.001, and p = 0.003, respectively). Areas under the ROC curve were found to be as follows: LDL-C to lymphocyte ratio: 0.774 (95% CI: 0.697-0.851), RDW-SD: 0.562 (95% CI: 0.456-0.669), and triglyceride level: 0.621 (95% CI: 0.531-0.732). The cut-off value of the LDL-C to lymphocyte ratio was 59.71 (sensitivity: 79.1%; specificity: 58.1%).

Conclusions

The LDL-C to lymphocyte ratio can be used as a marker to predict in-hospital mortality in patients with ACI. We recommend further studies to verify our findings.

## Introduction

Stroke is one of the major causes of disability and death in adults [1]. Acute cerebral infarction (ACI) accounts for approximately 80% of all strokes [2]. The mortality rates for ACI with large artery occlusion is still high [3]. Patient age and the severity of the stroke are known as the key determinants of poor prognosis [4]. Knowledge of prognostic factors can contribute to the management of stroke by clinicians. Therefore, investigators have been focusing on new and effective prognostic factors relating to ACI. In recent studies, hematological parameters, such as neutrophil to lymphocyte ratio [5], monocyte to high-density lipoprotein cholesterol (HDL-C) ratio [6], C-reactive protein to albumin ratio [7], red blood cell distribution width (RDW) [8], and the neutrophil to eosinophil ratio [9], were found to be associated with the prognosis of ACI.

The increase in low-density lipoprotein cholesterol (LDL-C) levels is known to be a risk factor for ischemic stroke [10,11]. Moreover, high LDL-C levels are associated with clinical deterioration in patients with ACI [12]. In recent years, studies have found that during the acute period, blood lymphocyte counts of patients with ACI who died were lower than those of patients who survived [8]. Therefore, the LDL-C to lymphocyte ratio can be a better and different expression of the increased LDL-C level and reduced lymphocyte count in the blood. In light of this information, the LDL-C to lymphocyte ratio could predict the in-hospital mortality of patients with ACI. To the best of our knowledge, the LDL-C to lymphocyte ratio in ACI has never been investigated. Hence, in this study, we aimed to investigate the LDL-C to lymphocyte ratio in ACI.

## Materials and methods

Patients who were treated for the diagnosis of ACI and underwent subsequent follow-ups at the Neurology Department of Aksaray University Training and Research Hospital between January 2015 and December 2018 were retrospectively examined. Patients who presented to our emergency room within the first 24 hours from the onset of symptoms were regarded as ACI, and the study was conducted with these patients. The subjects included in the study were divided into two groups. The first group comprised those who died from ACI that developed due to a detected cerebrovascular occlusion, while the second group comprised those who did not die from ACI (control group). Only those patients who were older than 18 years were included in the study. Subjects whose symptoms started more than 24 hours before the emergency room visit; those who were taking immunosuppressive drugs; those with an indication for mechanical thrombectomy and therefore referred; those with hematological disorders, liver and kidney disease, hemorrhagic infarction, electrolyte imbalance; and those who were under 18 years of age as well as those with missing data were excluded from the study. The study was conducted with laboratory data of patients obtained within the first 24 hours following the onset of symptoms.

Initially, medical history was obtained from patients who were brought to our hospital with a preliminary diagnosis of stroke or from their relatives. Subsequently, patients’ vital signs (body temperature, blood pressure, oxygen saturation, pulse, etc.), as well as finger stick blood glucose levels, were measured. Neurological evaluation was performed, while blood samples were taken for hemogram, international normalized ratio, activated partial thromboplastin time, and biochemical evaluation. For parenchymal imaging of the brain, CT of the brain or MRI of the brain was performed. In addition, computed tomography angiography of the brain or magnetic resonance angiography of the brain was performed for vascular imaging of the brain. Consequently, the clogged arteries of the patients were determined and the treatment to be performed was quickly planned accordingly. Patients with mechanical thrombectomy indications were referred. Other patients received treatment according to the most recent stroke guidelines [13]. Of these, 14 patients who met the intravenous (IV) thrombolytic therapy criteria were given IV thrombolytic therapy.

Peripheral venous blood samples were taken from all patients and centrifuged, and then, blood cell count analyses were performed using an autoanalyzer (Sysmex XN-1000 Hematology Analyzer; Sysmex Corporation, Kobe, Japan) in the hematology laboratory of our institution [9]. LDL-C to lymphocyte ratio was calculated by simply dividing the LDL-C amount by the lymphocyte count.

Information on clinical and laboratory findings, results of the parenchymal and vascular imaging of the brain, risk factors, and other demographic characteristics of the patients registered in our database were retrieved and saved for statistical analysis. The study was approved by the ethics committee of our institution and was carried out in accordance with the Declaration of Helsinki.

Statistical analysis

Results were presented as mean ± standard deviation for normally distributed data and as median (minimum-maximum) for abnormally distributed data. To investigate the distribution pattern of the data, the Kolmogorov-Smirnov normality test was used. Only red blood cell (RBC) data distributed normally, and hence were compared using Student's independent samples T-test; other blood test parameters did not distribute normally, and hence were compared using the Mann-Whitney U test. To compare the gender distribution of the groups, we used the chi-square test. To assess the predictive value of variables, the receiver operating characteristics (ROC) curve analysis test was used [9,14]. If the area under the ROC curve was 0.5, the model did not discriminate; if it was 0.5-0.7, the model had poor to fair discrimination; if it was 0.7-0.8, the model had acceptable discrimination; if it was was between 0.8-0.9, the model had excellent discrimination; a value of 0.9-1.0 was a very rare outcome. For statistical analysis of all data, we used SPSS Statistics version 23.0 software for Windows (IBM, Armonk, NY). A p-value of less than 0.05 was considered statistically significant.

## Results

A total of 172 patients with ACI were eligible for the study. The group without hospital mortality (survivors) consisted of 129 patients [67 males and 62 females, median age: 69 years (range: 22-93)] and the group with hospital mortality (non-survivors) consisted of 43 patients [15 males and 28 females, median age: 78 years (range: 58-99)]. The groups were gender-matched (p = 0.052), but the median age of the patients with hospital mortality was significantly higher compared to those without mortality (p: <0.001).

The comparison of blood parameters between the groups (survivors and non-survivors) is presented in Table 1. According to the Student's T-test, the RBC value did not significantly differ between the groups (p = 0.109). The Mann-Whitney U test revealed that the median total cholesterol, neutrophil to HDL-C ratio, monocyte to HDL-C ratio, white blood cell (WBC), RDW-CV, mean corpuscular volume and platelet values did not significantly differ between the patients with and without hospital mortality (p = 0.116, p = 0.230, p = 0.690, p = 0.844, p = 0.222, p = 0.353, and p = 0.513, respectively). However, the median triglyceride, LDL-C to lymphocyte ratio, and RDW-SD values were significantly higher in patients with in-hospital mortality, compared with the survivors (p = 0.017, p: <0.001 and p = 0.003, respectively).

**Table 1:** Comparison of blood parameters between survivor and non-survivor groups RBC: red blood cell; LDL: low-density lipoprotein; HDL: high-density lipoprotein; WBC: white blood cell; RDW: red blood cell distribution width; MCV: mean corpuscular volume

Parameters	Survivors (those without in-hospital mortality)	Non-survivors (those with in-hospital mortality)	P-value
RBC (10^12^/L)	4.86 ± 0.67	4.67 ± 0.64	0.109
Total cholesterol (mg/dL)	178 (111-327)	195 (131-382)	0.116
Triglyceride (mg/dL)	128 (56-366)	160 (70-322)	0.017
LDL/lymphocyte	54.48 (12.26-156.06)	89.19 (41.32-202.39)	<0.001
Neutrophil/HDL	0.146 (0.047-0.57)	0.126 (0.057-0.56)	0.230
Monocyte/HDL	0.015 (0.001-0.087)	0.013 (0.003-0.047)	0.690
WBC (10^9^/L)	8.7 (4.13-19.03)	8.71 (5.26-22.91)	0.844
RDW-CV (%)	13.3 (10.9-26)	13.8 (10.3-23.5)	0.222
RDW-SD (fL)	43.4 (27.9-70)	45.1 (38.1-57.6)	0.003
MCV (fL)	87.3 (60.3-99.6)	89.6 (68-102.6)	0.353
Platelet (10^9^/L)	227 (96-518)	219 (111-408)	0.513

**Figure 1:**
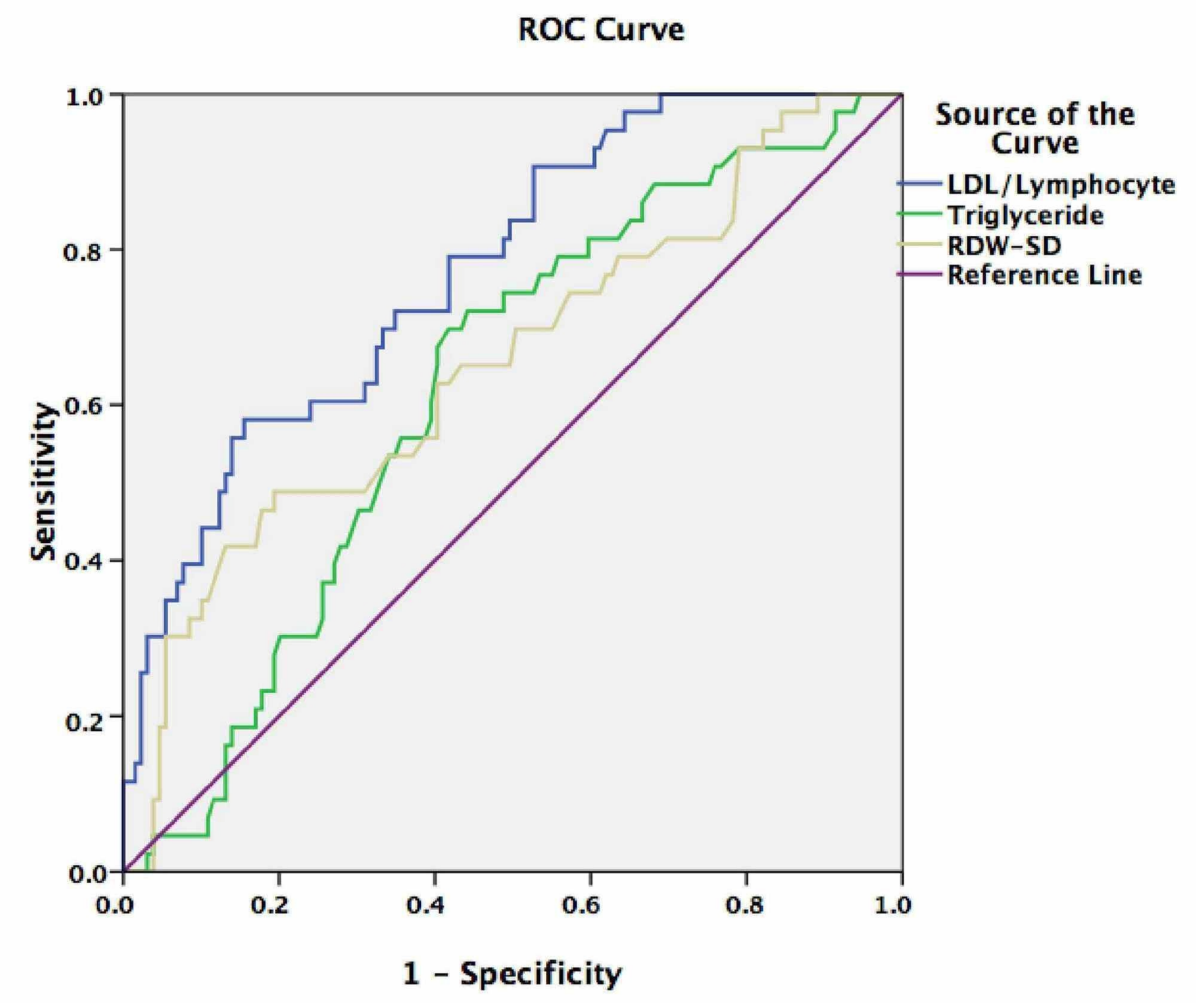
Receiver operating characteristic (ROC) curve representing the predictive value of the low-density lipoprotein cholesterol (LDL-C) to lymphocyte ratio, red blood cell distribution width-standard deviation (RDW-SD), and triglyceride level for mortality

Figure 1 shows the ROC curve representing the predictive value of LDL-C to lymphocyte ratio, RDW-SD, and triglyceride, for mortality. The areas under curve were as follows: LDL-C to lymphocyte ratio: 0.774 (95% CI: 0.697-0.851), RDW-SD: 0.562 (95% CI: 0.456-0.669), and triglyceride: 0.621 (95% CI: 0.531-0.732). The cut-off value of LDL-C to lymphocyte ratio indicating mortality in patients with ACI was found to be 59.71 (sensitivity: 79.1%; specificity: 58.1%).

## Discussion

This study showed that an early high LDL-C to lymphocyte ratio is an independent predictor of in-hospital mortality in patients with ACI. In the present study, we used the laboratory data within the first 24 hours following the onset of the patients’ symptoms to determine whether they are early predictors of mortality. To the best of our knowledge, this is the first study investigating the LDL-C to lymphocyte ratio in ACI.

Although it has been revealed in recent years that some inflammatory cells, such as neutrophils [15] and pro-inflammatory cytokines [16], play an important role in secondary brain damage in ACI, the pathophysiological mechanisms of secondary brain damage are still not clear yet. Therefore, this issue remains a focus for neuroscientists.

Secondary brain damage in ACI occurs with a chain of pathophysiological events involving a number of inflammatory cells. Neutrophils have particularly played an important role in this damage. Cell necrosis begins soon after the stroke, followed by increased reactive oxygen species in this area, and activation of microglia and astrocytes begins with the chemokines and cytokines released. Overactivation of microglia and astrocytes leads to the release of chemokines, pro-inflammatory cytokines, and free oxygen species [16,17]. They, consequently, cause the passage of neutrophils produced in peripheral blood to the ischemic brain region approximately four to six hours after ACI [16,17]. Increased neutrophils in and around the ischemic region of the brain contribute to oversecretion of pro-inflammatory cytokines, overproduction of reactive oxygen species, overregulation of matrix metalloproteinase-9, and macrophage activation [16-18]. The endpoint reached with this chain of pathophysiological events is secondary brain damage caused by the disruption of the blood-brain barrier, neuronal death, increased edema, and enlargement of the ischemic area [16-18]. In ACI, the neutrophil count increases, while the lymphocyte count decreases [5,19]. Although a correlation exists between high LDL-C levels and clinical deterioration [12] and long-term mortality [20] in patients with ACI, its relationship with mortality is not clear [21]. In light of this information, we believe that the LDL-C to lymphocyte ratio may represent a better and different expression of the increase in the amount of LDL-C and the decrease in lymphocyte count in the blood. The LDL-C to lymphocyte ratio is important as it has a better predictive value than RDW in terms of its potential to predict the mortality of ACI in the present study. Finally, due to these significant results obtained in the present study, we believe that the LDL-C to lymphocyte ratio can be used as a marker that can predict in-hospital mortality in patients with ACI. We believe that further studies conducted on this subject in the future can verify this. In the present study, we found that in addition to the LDL-C to lymphocyte ratio, RDW-SD predicts mortality in patients with ACI. This result is consistent with that of previous studies [5,8].

Although this study was conducted to determine whether the early LDL-C to lymphocyte ratio has a predictive value in the in-hospital mortality of patients with ACI and to emphasize the importance of this in ACI, it also has some limitations. They include the relatively low number of patients we included in the study, the absence of comparison of the laboratory parameters between acute and subacute periods of the patients, and the single-center and retrospective nature of the study.

## Conclusions

Based on our findings, an early high LDL-C-to-lymphocyte ratio can predict the in-hospital mortality of patients with ACI. It may even have a better predictive value than RDW-SD. Further studies on LDL-C to lymphocyte ratio with a higher number of patients are needed to implement it as a new predictive marker in ACI.
